# A metabolism-associated gene signature for prognosis prediction of hepatocellular carcinoma

**DOI:** 10.3389/fmolb.2022.988323

**Published:** 2022-09-30

**Authors:** Yilin Tian, Jing Lu, Yongxia Qiao

**Affiliations:** ^1^ School of Public Health, Shanghai Jiaotong University School of Medicine, Shanghai, China; ^2^ Shanghai Institute of Hematology, Ruijin Hospital, Shanghai Jiao Tong University School of Medicine, Shanghai, China

**Keywords:** hepatocellular carcinoma, metabolic pathways, metabolism-associated genes, LASSO model, prognosis signature

## Abstract

Hepatocellular carcinoma (HCC), the most frequently occurring type of cancer, is strongly associated with metabolic disorders. In this study, we aimed to characterize the metabolic features of HCC and normal tissue adjacent to the tumor (NAT). By using samples from The Cancer Genome Atlas (TCGA) liver cancer cohort and comparing 85 well-defined metabolic pathways obtained from the Kyoto Encyclopedia of Genes and Genomes (KEGG), 70 and 7 pathways were found to be significantly downregulated and upregulated, respectively, in HCC, revealing that tumor tissue lacks the ability to maintain normal metabolic levels. Through unsupervised hierarchical clustering of metabolic pathways, we found that metabolic heterogeneity correlated with prognosis in HCC samples. Thus, using the least absolute shrinkage and selection operator (LASSO) and filtering independent prognostic genes by the Cox proportional hazards model, a six-gene-based metabolic score model was constructed to enable HCC classification. This model showed that high expression of *LDHA* and *CHAC2* was associated with an unfavorable prognosis but that high *ADPGK*, *GOT2*, *MTHFS,* and *FTCD* expression was associated with a favorable prognosis. Patients with higher metabolic scores had poor prognoses (*p* value = 2.19e-11, hazard ratio = 3.767, 95% CI = 2.555–5.555). By associating the score level with clinical features and genomic alterations, it was found that NAT had the lowest metabolic score and HCC with tumor stage III/IV the highest. qRT‒PCR results for HCC patients also revealed that tumor samples had higher score levels than NAT. Regarding genetic alterations, patients with higher metabolic scores had more *TP53* gene mutations than those with lower metabolic scores (*p* value = 8.383e-05). Validation of this metabolic score model was performed using another two independent HCC cohorts from the Gene Expression Omnibus (GEO) repository and other TCGA datasets and achieved good performance, suggesting that this model may be used as a reliable tool for predicting the prognosis of HCC patients.

## Introduction

Hepatocellular carcinoma (HCC) is the most common type of liver cancer and the 4^th^ leading cause of cancer death worldwide (Yu and Schwabe, 2017; Villanueva, 2019). As a key metabolic organ in the body, the liver plays a key role in energy metabolism and detoxification. When tumor cells become malignant and migrate to the liver, they can destroy the metabolic functional base of the liver and cause jaundice, pain, and weight loss, which might ultimately lead to death (Phan et al., 2014; Anwanwan et al., 2020). Previously reported risk factors for HCC include viral infection, such as with hepatitis B virus (HBV), nonalcoholic fatty liver disease, smoking, diabetes, and alcohol-induced cirrhosis (Morgan et al., 2004; Zoller and Tilg, 2016). Due to tumor heterogeneity and multiple risk factors, the molecular mechanisms of HCC onset and progression are still not clearly understood (Ogunwobi et al., 2019).

Abnormal tumor cell metabolism has been reported to deeply participate in the pathogenesis of tumor growth and shape the tumor microenvironment (TME) (Reina-Campos et al., 2017). As a hallmark of cancer, metabolic alterations can be categorized into different types (Gong et al., 2020), including amino acid metabolism, carbohydrate metabolism, energy metabolism, glycan biosynthesis and metabolism, lipid metabolism, and cofactor and vitamin metabolism. Previously reported studies on metabolism have revealed that metabolic pathways and metabolites play an important role in hepatocarcinogenesis in liver cancer (Perumpail et al., 2015; Gingold et al., 2018; Alannan et al., 2020). For example, dysregulation of energy metabolism can enable tumor cells to produce more adenosine triphosphate (ATP) to support tumor proliferation and migration (DeBerardinis and Chandel, 2016; Chen et al., 2020), and extramitochondrial fatty acid oxidation is relevant to the regulation of neoplastic cell growth of HCC (Ockner et al., 1993). Therefore, characterization of the metabolic features of HCC is important for investigating its hepatocarcinogenesis mechanism and providing therapeutic targets.

**FIGURE 1:**
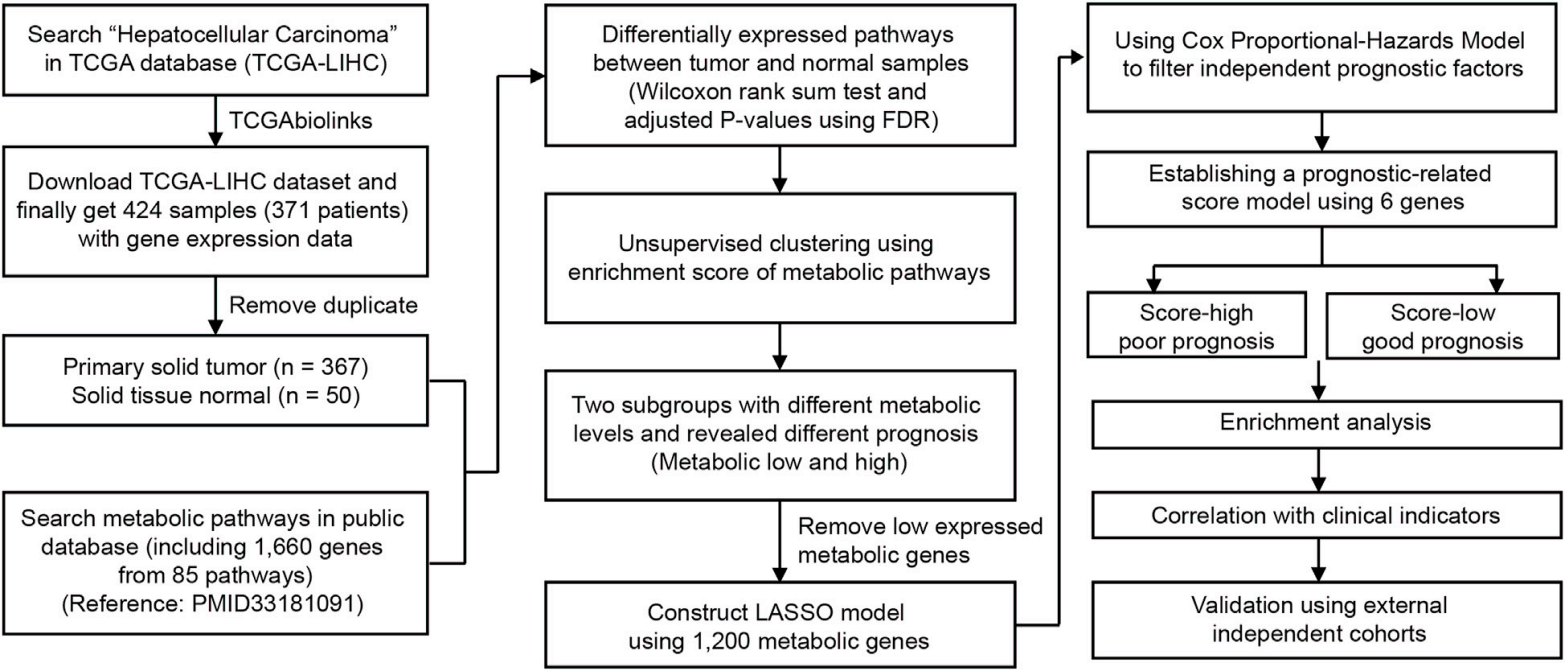
Overview of the analyzing workflow and establishment of the metabolic model of HCC in this study.

In this study, we aimed to deeply explore the metabolic features and investigate the tumor heterogeneity of HCC. To better interpret metabolic pathways, we collected 85 well-established metabolic gene sets (one pathway with only one gene not included) from KEGG (Gong et al., 2020) and summarized them into nine major types. To collect HCC data, 424 HCC and NAT samples with RNA sequencing data were obtained from The Cancer Genome Atlas (TCGA) (Figure 1). After the removal of duplicates, 367 primary solid tumor and 50 normal tissue adjacent to the tumor (NAT) samples were used for further analysis (Figure 1). The relationship between metabolic pathway scores and prognosis and other clinical characteristics was evaluated. Next, six genes from among 1,200 metabolic genes were selected to construct a prognostic-related metabolic score model using the least absolute shrinkage and selection operator (LASSO), which was applied for HCC classification.

## Materials and methods

### Data preprocessing

Bulk RNA-seq and clinical data of HCC used for survival analysis and prognostic model construction were downloaded from the TCGA database (https://portal.gdc.cancer.gov/) under accession TCGA-LIHC (liver hepatocellular carcinoma). Only primary solid tumor and normal tissue adjacent to the tumor (NAT) samples were enrolled for analysis. Patients without survival information were removed from further evaluation of the model. Both TCGA datasets and clinical information were downloaded using TGCAbiolinks (Colaprico et al., 2016). External independent HCC cohorts were obtained from Gene Expression Omnibus (GEO, http://www.ncbi.nlm.nih.gov/geo/) under accession IDs GSE14520 and GSE76427. The expression data and clinical information of these two HCC cohorts were downloaded using GEOquery (Davis and Meltzer, 2007) or obtained from the supplementary data of published research works (Roessler et al., 2010; Grinchuk et al., 2018). For RNA sequencing data, the fragments per kilobase per million mapped fragments (FPKM) value was used to construct the model and calculate the metabolic score.

### Identification of differentially expressed metabolic genes/pathways in HCC

Metabolic gene sets were obtained from previously published research works (Gong et al., 2020) and collected from the KEGG database. Metabolic pathways defined by only one gene were excluded from further analysis. Thus, only 85 metabolic pathways (including 1,660 genes) were used in the analysis. We then classified these 85 metabolic pathways into nine major types: amino acid metabolism, carbohydrate metabolism, energy metabolism, glycan biosynthesis and metabolism, lipid metabolism, metabolism of cofactors and vitamins, nucleotide metabolism, xenobiotics biodegradation and metabolism, and others. Pathway names, major types, and genes in the metabolic pathways are listed in Supplementary Table S1. We used an enrichment score to evaluate the expression level of each metabolic pathway. The enrichment score of these metabolic pathways was calculated using single-sample Gene Set Enrichment Analysis (ssGSAE) in the R package GSVA (Hanzelmann et al., 2013). Differential analysis between tumors and NATs was calculated using the mean value of the enrichment score of each type. *p* values were calculated using the Wilcoxon rank-sum test and adjusted using Benjamini and Hochberg (FDR). The significance level of the metabolic pathway score was set as *FDR* < 0.05. Significance of the metabolic pathways between tumor and normal samples are listed in Supplementary Table S2. For gene level analysis, differentially expressed metabolic genes were calculated using R package limma (Ritchie et al., 2015). The significance level was defined by an adjusted *p* value < 0.05 and log_2_ fold change > 1 (fold change > 2).

### Construction of the metabolic score model using LASSO

For the filtration of 1,660 metabolism-related genes, we first removed genes with low expression and retained those with detected expression in all HCC samples. A total of 1,200 genes were used to construct the model. The LASSO model was used for the next-step filtration of genes, which was implemented in the R package glmnet (v4.0.2). To evaluate the variability and reproducibility of the estimates produced by the LASSO, we repeated the regression fitting process and calculated the best lambda to reduce the error rate by 10-fold cross-validation. Then, 23 genes with nonzero coefficient estimates were retained. To further reduce genes and identify genes correlating with prognosis, multivariate Cox proportional hazards regression was performed to estimate the coefficient in survival analysis; independent prognostic factors (genes with *p* values less than 0.05) were kept for the next step of LASSO. Finally, six genes were selected, and the metabolic score was determined. The median value of the metabolic score was used as the cutoff to separate HCC data into two groups. Basic information on HCC patients in TCGA-LIHC patients, including the metabolic score, is listed in Supplementary Table S3. Patients were grouped into metabolic score-low and -high groups. The R package forestplot was used for presentation of the results for TCGA-LIHC, HCC cohorts obtained from GEO, and other TCGA cancer datasets. The Kaplan–Meier method was used to generate survival curves for the score-low and -high groups in each dataset, and the log-rank test was used to determine the statistical significance of differences. The hazard ratios for univariate analysis were calculated using the Cox proportional hazards regression model. A multivariate Cox regression model was used to determine independent prognostic factors using the survival package.

### RNA isolation and qRT‒PCR analysis

The human hepatoma cell lines BEL-7402 and BEL-7404 were established from clinical liver cancer surgical specimens (Chen et al., 1980). Total RNA was isolated using TRIzol reagent (Invitrogen, United States) following the manufacturer’s protocol and quantified by nanodrop 8,000. In brief, cells were lysed with TRIzol reagent, and chloroform was then added. After centrifugation, the aqueous phase was collected and mixed with isopropanol before centrifugation. RNA was dissolved in RNase-free water. For analysis of mRNA expression, 1 µg of RNA was converted into cDNA using the PrimeScript™ RT Reagent Kit (Invitrogen, United States). Quantitative real-time polymerase chain reaction (PCR) using ChamQ Universal SYBR® qPCR Master Mix (Vazyme, China) was performed on a QuantStudio5 Real-time PCR system (Applied Biosystems). The quantitative PCR primer sequences of the metabolic genes and the endogenous control GAPDH are listed in Supplementary Table S4.

### Functional enrichment analysis and mutation analysis

The clusterProfiler (Yu et al., 2012) R package was used to perform functional enrichment analysis on differentially expressed genes between the metabolic score groups. Gene sets used in the enrichment analysis were downloaded from the Molecular Signatures Database (MSigDB, v7.4) of the Broad Institute (Liberzon et al., 2015). The gene sets were downloaded from MSigDB, including HALLMARK gene sets (H) and KEGG gene sets (C2). HALLMARK and Kyoto Encyclopedia of Genes and Genomes (KEGG) terms were used for functional enrichment of genes with a strict cutoff of *FDR* < 0.05. For mutation analysis, mutations in HCC samples from TCGA were obtained from the cBio cancer genomics portal (cBioPortal, https://www.cbioportal.org/) (Cerami et al., 2012). The mutation profiles of low and high metabolic scores were visualized using the R package maftools (Mayakonda et al., 2018).

### Statistical and survival analysis

The Wilcoxon rank-sum test was used for comparisons of the two groups. Correlation coefficients were computed by Spearman and distance correlation analyses. Two-sided Fisher exact tests were used to analyze contingency tables. To identify significant genes in differential gene analysis, we applied the Benjamini–Hochberg (alias FDR) method to convert the *p* value to *FDR*. The *p* values were two-sided, and less than 0.05 was considered statistically significant. For survival analysis, Kaplan–Meier and log-rank tests were performed using the survival (https://CRAN.R-project.org/package=survival) and survminer (https://CRAN.R-project.org/package=survminer) packages. For specific genes, patients were divided into high- or low-expression groups according to the median expression of the gene, and a *p* value < 0.05 was considered to denote significance (Zeng et al., 2019). All heatmaps were generated by the R package pheatmap (https://github.com/raivokolde/pheatmap).

## Results

### Metabolic disorders of HCC

**FIGURE 2:**
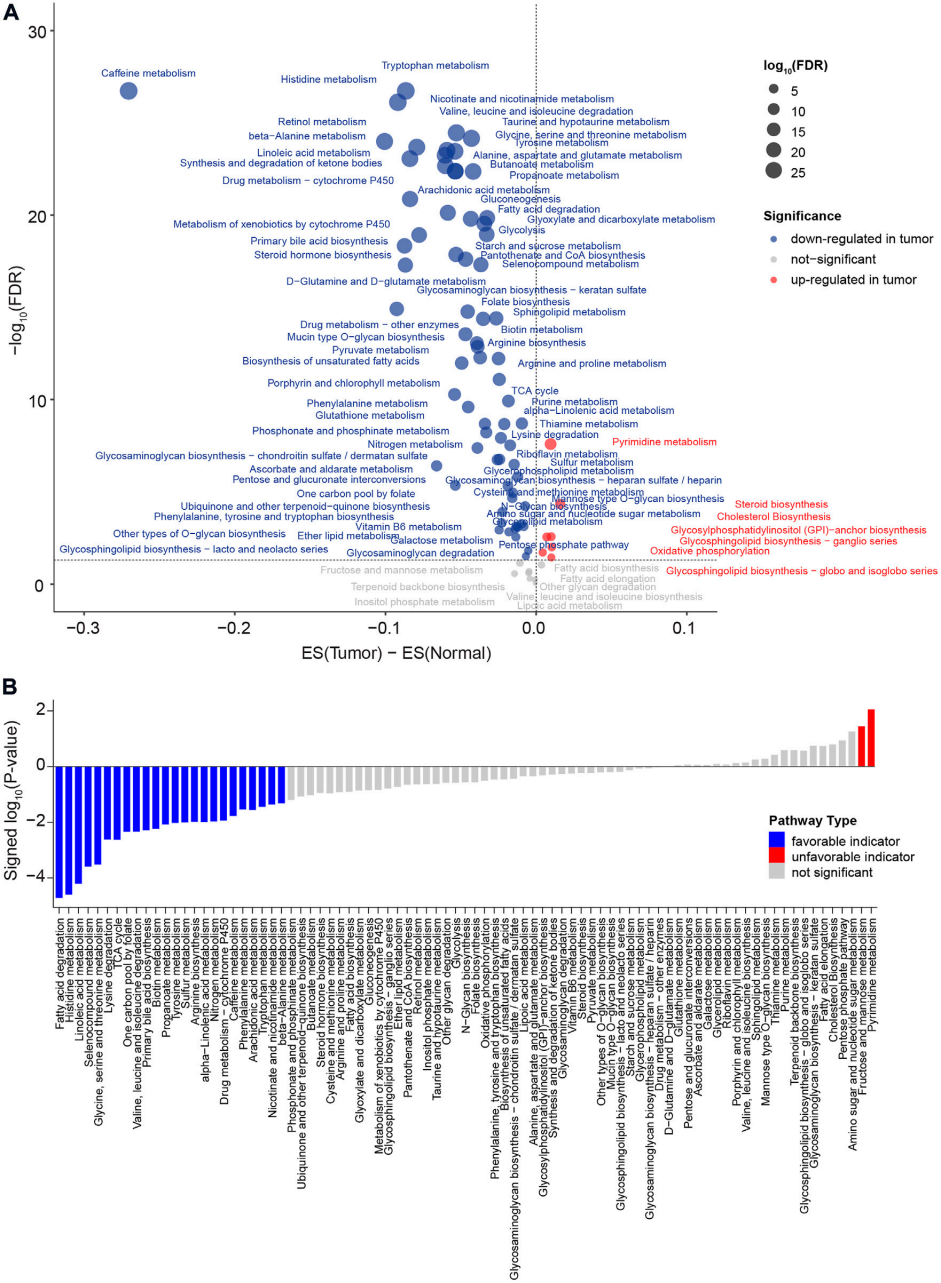
Comparison of metabolic pathways between HCC and NAT and characterization of prognosis of metabolic pathway levels. **(A)** Dot plot of the dysregulated metabolic pathways between HCC and NAT. The X-axis is the difference between the mean enrichment score of HCC and NAT, while the Y-axis is the log_10_ transformed FDR. The red dot represents the significant upregulated metabolic pathways in HCC, and the blue dot represents the significant downregulated metabolic pathways in HCC. *p* values were calculated using the Wilcoxon rank-sum test and adjusted using FDR. **(B)** Bar plot of significant levels of metabolic pathways with overall survival analysis. The 85 metabolic pathways were ordered by the signed log_10_ *p* value. For favorable indicators (higher expression, favorable prognosis), the bars are colored in blue (*p* value < 0.05). The unfavorable indicators are colored in red. *p* values were calculated log-rank test.

A flow chart was used to illustrate the analysis workflow of this project (Figure 1). After removing duplicate samples, 367 patients were diagnosed with HCC, and 50 NAT samples were used for analysis. We first calculated potential risk factors for HCC using clinical overall survival data, which are shown in Supplementary Figure S1. For HCC, the American Joint Committee on Cancer (AJCC) stage of the tumor, which consisted of the primary tumor (AJCC_T) and regional lymph nodes (AJCC_N) and distant metastasis (AJCC_M), was the most important prognostic factor (Supplementary Figure S1). Then, we calculated the expression levels of metabolic pathways of HCC and NAT and associated them with overall survival to identify prognosis-related pathways. To evaluate the metabolic level of each sample, the enrichment score of each pathway was calculated and then compared between HCC and NAT using the Wilcoxon rank-sum test (Figure 2A). In total, 70 (82.4%) pathways were significantly downregulated, and 7 pathways (8.24%) were significantly upregulated in HCC, revealing a lack of ability to maintain normal metabolic levels in tumors (Figure 2A). Most (18/19, 94.7%) amino acid metabolism pathways, including tryptophan, histidine, glycine, serine, and threonine metabolism, were suppressed in HCC, suggesting that normal catabolism of amino acids was disturbed. Most metabolic pathways involved in normal liver functions, such as lipid and carbohydrate metabolisms, were downregulated in HCC. Regarding upregulated pathways, we noticed that pathways related to pyrimidine metabolism (required for cell proliferation) (Siddiqui and Ceppi, 2020), steroid and cholesterol biosynthesis (promote tumorigenesis) (Huang et al., 2020), and oxidative phosphorylation were significantly upregulated in tumors, indicating increased phosphorylation levels and malignant proliferation in HCC (Figure 2A). After associating with overall survival data, it was found that two pathways, pyrimidine metabolism (belonging to nucleotide metabolism) and fructose and mannose metabolism (belonging to carbohydrate metabolism), were unfavorable indicators; 24 pathways, including fatty acid degradation, histidine metabolism, linoleic acid metabolism, selenocompound metabolism, glycine, serine, and threonine metabolism, lysine degradation, and TCA cycle, were favorable indicators of HCC (Figure 2B and Supplementary Figure S2). These results suggest that metabolic disorders are prevalent in tumor tissue and may be used as prognostic indicators for the overall survival of patients.

### Construction of a LASSO-Cox-based model to predict the prognosis of HCC patients

**FIGURE 3:**
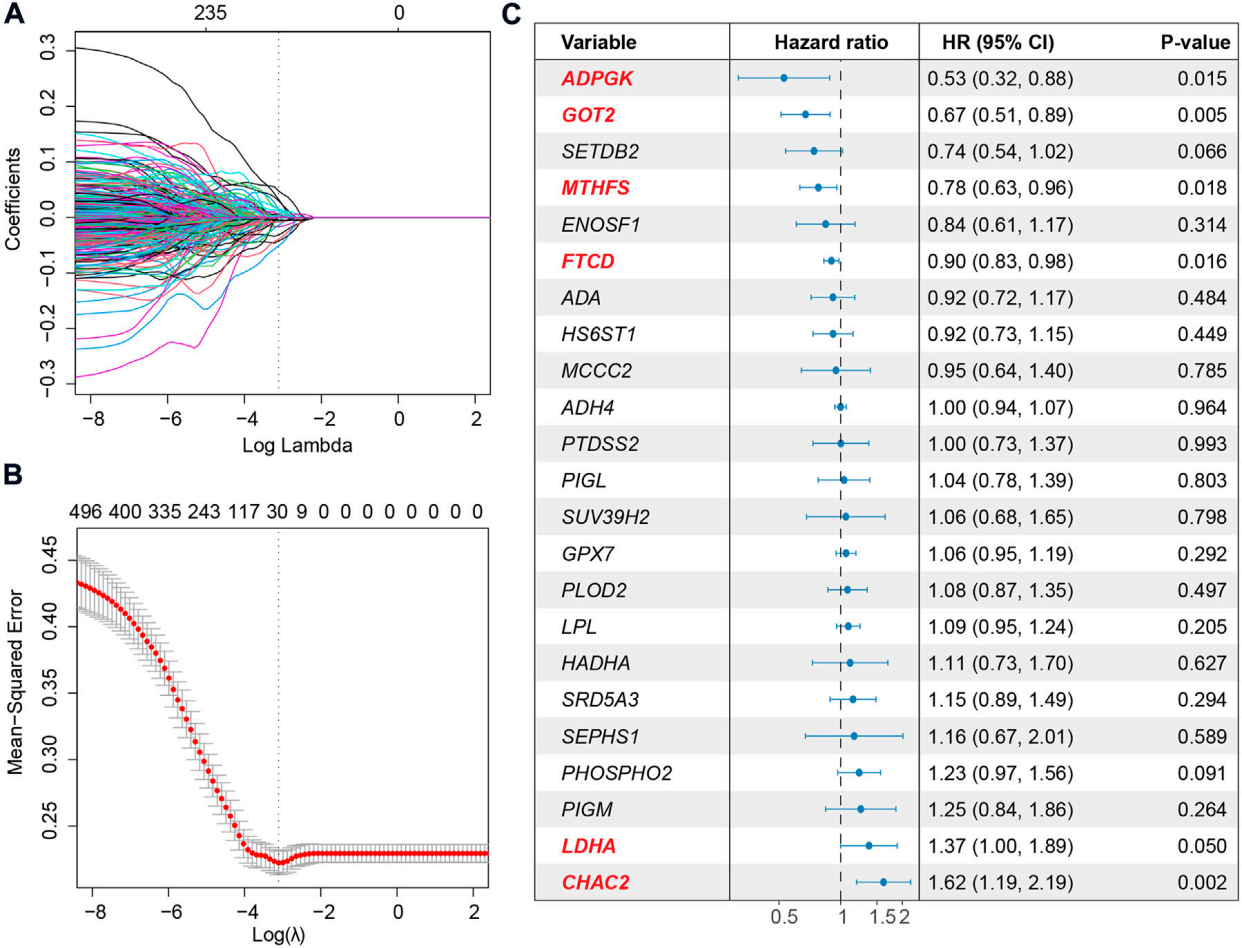
Extraction of the prognostic signature and identification of final metabolic-related genes to establish the metabolic model. **(A)** Coefficient selection and variable screening of LASSO. The minimum mean cross-validated error of λ is selected. The lower X-axis represents the lambda value, and the upper X-axis scale represents the number of metabolic genes in the LASSO model. **(B)** Cross-validation in the LASSO model to select the tuning parameter. The X-axis represents the log (lambda) value, and the Y-axis represents the partial likelihood deviance. **(C)** Forest plots of multivariate analysis showing the six genes (*ADPGK*, *GOT2*, *MTHFS*, *FTCD*, *LDHA,* and *CHAC2*) as independent prognostic factors of overall survival of HCC patients.

To deeply investigate the correlation between metabolic pathways and the overall survival of HCC, we performed unsupervised hierarchical clustering using metabolic pathway scores calculated for the 367 HCC patients. The patients could be divided into two clusters, one with overexpression of the most metabolic pathways and another with a lower expression level, based on the profile (Supplementary Figure S3A). Survival analysis revealed that patients with a more active metabolic level might have a favorable prognosis (Supplementary Figure S3B). Differentially expressed genes between the two clusters showed differences at the metabolic gene level (Supplementary Figure S3C). Therefore, for a better interpretation of the metabolic signature of HCC, we used LASSO to establish a metabolic score model and presented the relationship between metabolism and overall survival. Metabolic genes expressed at lower levels or not were filtered, and 1,200 genes were used for further analysis. Then, LASSO was used to narrow down the number of genes by giving a zero to the estimated coefficient of these genes (Figure 3A and Figure 3B). The model with a minimum lambda of 0.0501 was selected, and a total of 23 genes were identified. We then used the Cox proportional hazards model to filter independent prognostic factors. Six genes (*ADPGK*, *GOT2*, *MTHFS*, *FTCD*, *LDHA,* and *CHAC2*) were identified as independent prognostic factors using univariate and multivariate survival analyses (Figure 3C and Supplementary Figure S4). Finally, we constructed a six-gene-based metabolic score model, which is shown as follows:

Metabolic score = *ADPGK* * (-0.3254) + *GOT2* * (-0.2473) + *MTHFS* * (-0.1798) + *FTCD* * (-0.0717) + *LDHA* * 0.2449 + *CHAC2* * 0.3262.

**FIGURE 4:**
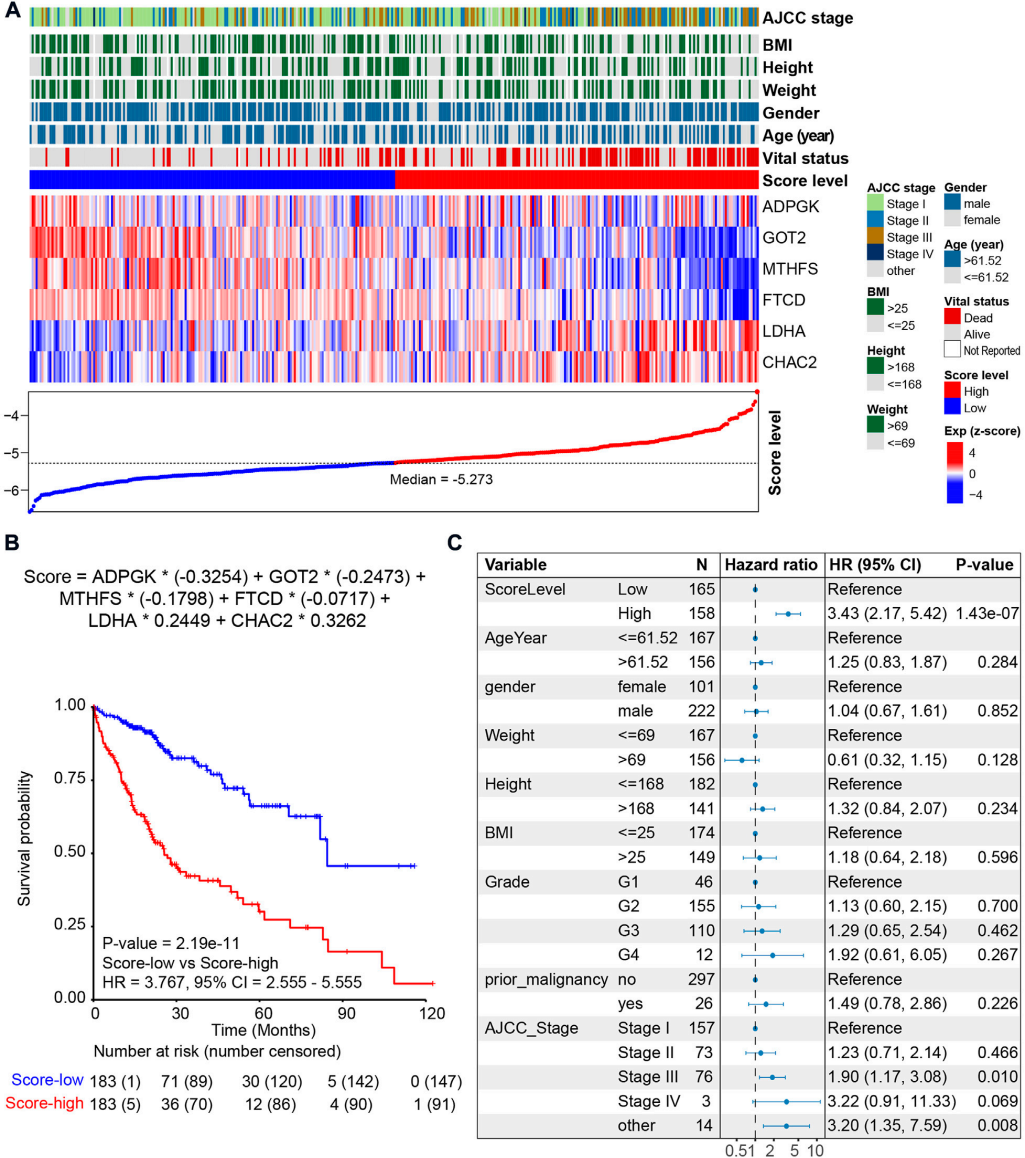
Prognosis of the metabolic score model in the HCC cohort. **(A)** LASSO model of the HCC cohort. Each column represents one patient. Patients are ordered by the metabolic score level. The upper panel shows the clinical feature of HCC patients, including AJCC stage, body mass index (BMI), height, weight, gender, age, vital status, and metabolic score. The middle panel shows the expression level of the six genes selected by LASSO. The lower panel shows the score level and the cutoff (median value -5.273) of HCC patients. **(B)** Kaplan–Meier curve comparing overall survival of metabolic score-low and -high. Patients are separated into two groups according to the median value (-5.273) of the metabolic score. *p* value is calculated using the log-rank test. **(C)** Forest plots of multivariate analysis showing the metabolic score as an independent prognostic factor of overall survival of HCC patients.

Among the six metabolic genes, *ADPGK* is an ADP-dependent glucokinase and catalyzes ADP-dependent phosphorylation of glucose, which is involved in gluconeogenesis/glycolysis in cancer progression and is upregulated in HCC tumor tissues (Ronimus and Morgan, 2004; Jing et al., 2020) (Supplementary Figure S4A). *GOT2* (glutamic-oxaloacetic transaminase 2) is a pyridoxal phosphate-dependent enzyme and plays a key role in amino acid metabolism (Stegen et al., 2020) and is upregulated in normal tissues (Supplementary Figure S4A). Regarding *MTHFS* (methenyltetrahydrofolate synthetase) and *FTCD* (formimidoyltransferase cyclodeaminase), both genes participate in the metabolism of cofactors and vitamins and are downregulated in HCC with a higher tumor stage (Matakidou et al., 2007; Wu et al., 2009; Love et al., 2012; Kanarek et al., 2018) (Supplementary Figure S4A). Our analysis of the cohort TCGA-HCC revealed that high expression of *ADPGK*, *GOT2*, *MTHFS*, and *FTCD* was associated with a favorable prognosis (Supplementary Figure S4B). *LDHA* (lactate dehydrogenase A) and *CHAC2* (ChaC glutathione-specific gamma-glutamylcyclotransferase 2) participate in amino acid metabolism. Previous studies on *LDHA* have reported that elevation of *LDHA* expression can promote the invasion and metastasis of tumors (Jin et al., 2017). *CHAC2* may act as a tumor suppressor in gastric and colorectal cancer (Liu et al., 2017). Univariate survival analysis also showed that high expression of *LDHA* and *CHAC2* was associated with an unfavorable prognosis, indicating that they could be considered biomarkers of HCC (Supplementary Figure S4B). Using the median value of metabolic score -5.273 as the cutoff, we calculated the metabolic score and found that the prognosis of patients with higher scores was poorer (Figure 4A and Figure 4B). The HR of the metabolic score was 3.767 (*p-value* = 2.19e-11, 95% CI = 2.555–5.555, Figure 4B); Supplementary Table S3 shows the metabolic scores for the 367 HCC patients. Multivariate survival analysis with age, sex, weight, height, prior malignancy, and AJCC stage also revealed the metabolic score as an independent prognostic indicator (Figure 4C).

### Association with clinical characteristics and genetic alterations

**FIGURE 5:**
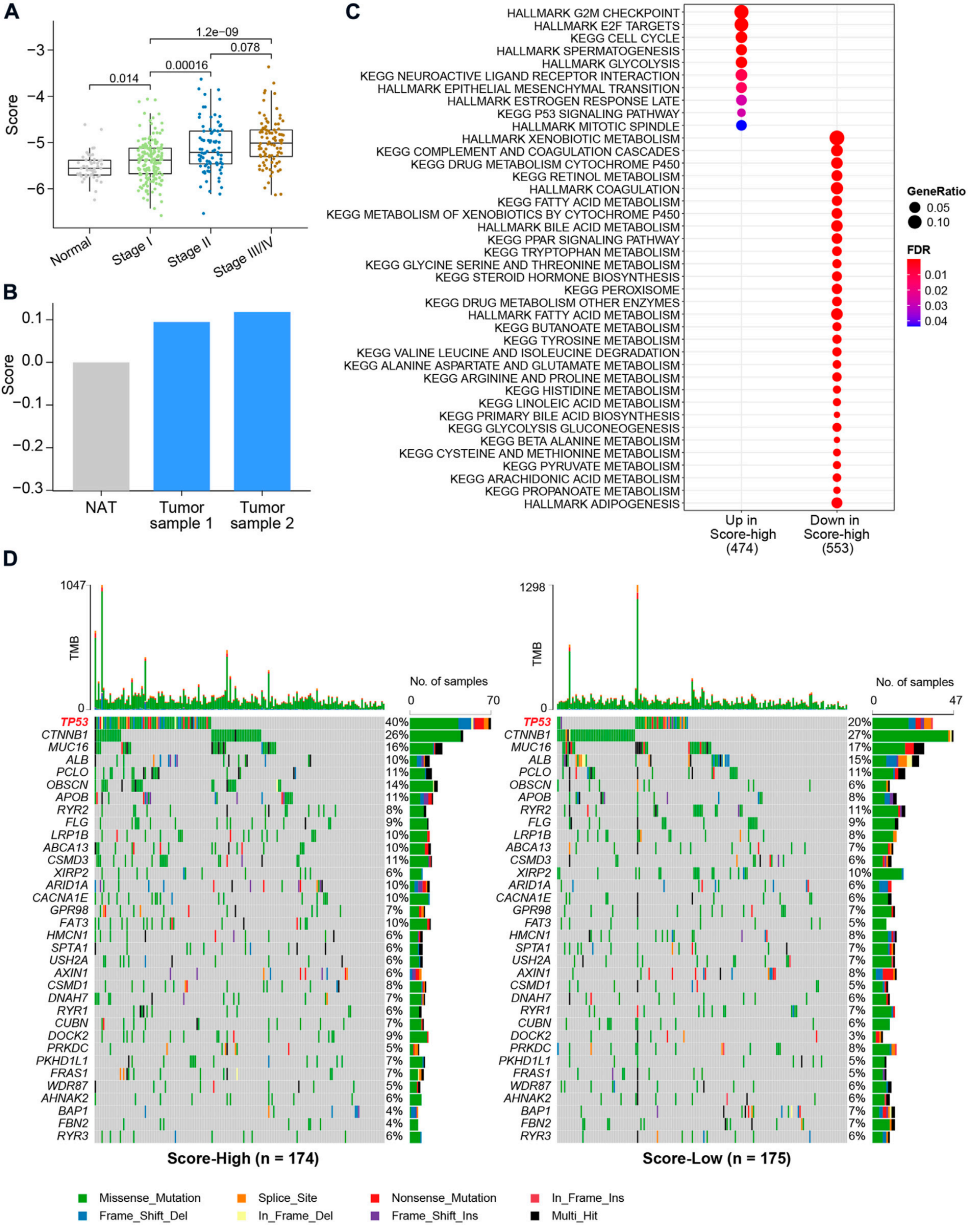
Clinical characteristics and genetic alterations associated with the metabolic score in HCC. **(A)** Boxplot of metabolic score in HCC and NAT. The X-axis shows the AJCC stages of HCC. *p* values were calculated using the Wilcoxon rank-sum test. **(B)** Bar plot of metabolic calculated using the qRT-PCR. **(C)** Enrichment analysis using differentially expressed genes between metabolic score-low and -high groups. **(D)** Oncoplot of the mutation profiles of metabolic score-low and -high groups. Gene with overall mutation frequency > 5% were selected for visualization.

We then associated the metabolic score with clinical data and genetic alterations and found the score level to be significantly lower in NAT (Figure 5A). The metabolic scores were highest for patients with AJCC stage III/IV (Figure 5A). Through qRT‒PCR, a higher HCC score was validated using two HCC tumor cell lines and one normal control cell (Figure 5B). After calculation of differentially expressed genes between the metabolic score-low and -high groups, we found genes significantly upregulated (FDR<0.05) in the high-score group to be enriched in cell proliferation pathways, such as the G2/M checkpoint, E2/F target, cell cycle, and epithelial–mesenchymal transition (EMT), and oncogenic pathways, such as the TP53 signaling pathway (Figure 5C). To further investigate the correlation between the six key metabolic genes and the role in affecting metabolic/oncogenic pathways in HCC, we performed protein–protein interaction networks functional enrichment analysis based on the STRING database (Szklarczyk et al., 2021). Interestingly, the results showed the direct pathways that correlated with the six genes, namely, the HIF-1 signaling pathway, pathways in cancer, metabolic pathways, WNT signaling pathway, JAK-STAT signaling pathway, and p53 signaling pathway (Supplementary Figure S5), revealing that the six genes played an important role in HCC (Supplementary Figure S5). Functional enrichment analysis also revealed upregulated genes in the low-score groups to be metabolism-related pathways, such as propanoate, arachidonic acid, and fatty acid metabolism, suggesting that this metabolic score model may be a valuable tool to evaluate metabolic disorders in HCC (Figure 5C). By comparing mutations between the two metabolic score groups, it was found that patients with higher scores harbored more *TP53* gene mutations (*p* value = 8.383e-05, Pearson’s chi-squared test, Figure 5D). However, there was no difference in tumor mutational burden (TMB) between the two groups, indicating that *TP53* gene mutations are a key factor contributing to metabolic disorders in HCC.

### Validation of the metabolic score model in external independent cohorts

**FIGURE 6:**
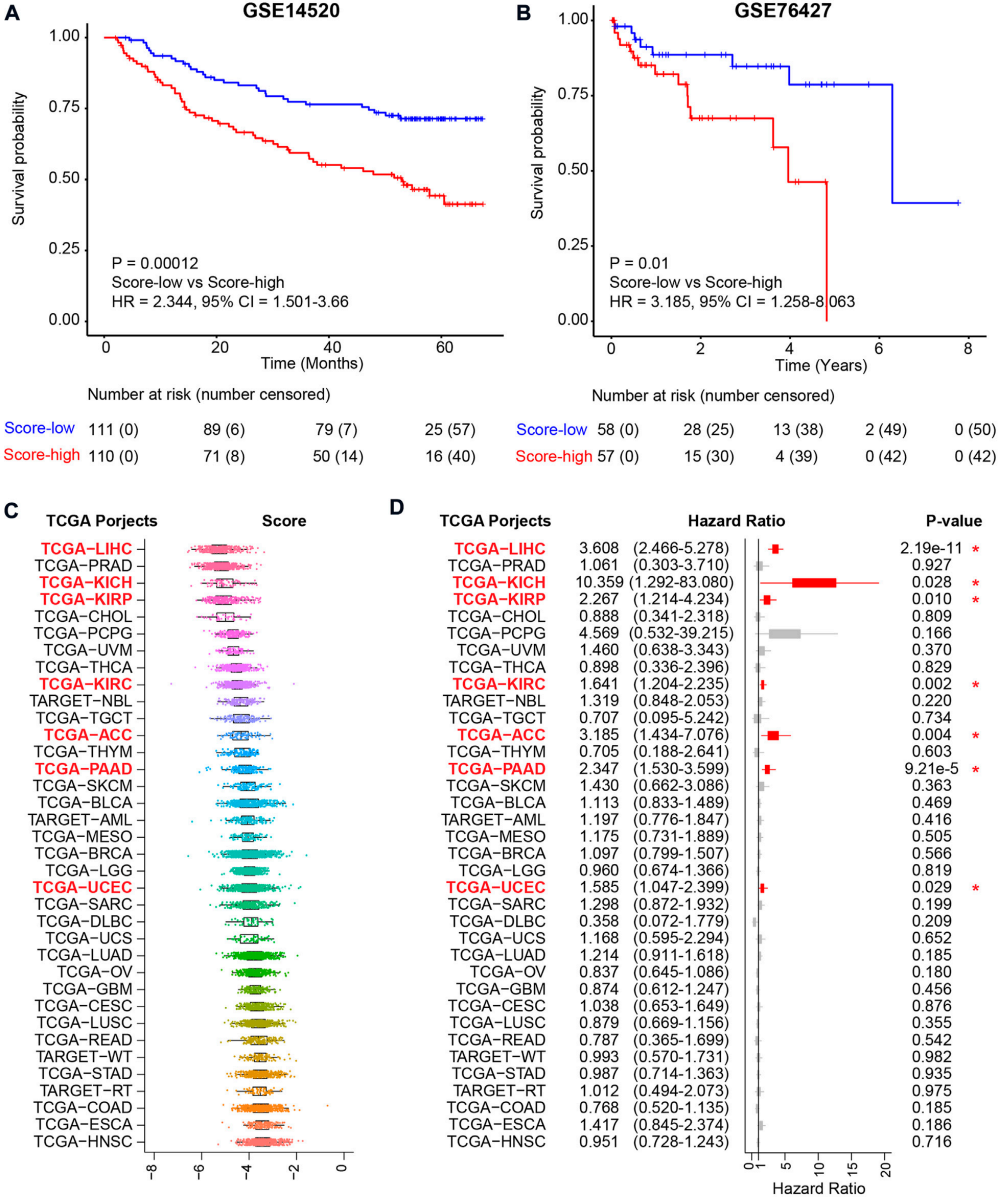
Validation of the metabolic score model in external independent HCC cohorts and TCGA projects. **(A)** Kaplan–Meier curve comparing overall survival of metabolic score-low and -high groups in the GSE14520 cohort. Patients are separated into two groups according to the median value of metabolic score in this cohort. *p* value is calculated using the log-rank test. **(B)** Kaplan–Meier curve comparing overall survival of metabolic score-low and -high groups in the GSE76427 cohort. **(C)** The metabolic score level across the 36 TCGA projects. Abbreviations of cancer types in TCGA projects: TCGA-LIHC: liver hepatocellular carcinoma, TCGA-PRAD: prostate adenocarcinoma, TCGA-KICH: kidney chromophobe, TCGA-KIRP: kidney renal papillary cell carcinoma, TCGA-CHOL: cholangiocarcinoma, TCGA-PCPG: pheochromocytoma and paraganglioma, TCGA-UVM: uveal melanoma, TCGA-THCA: thyroid carcinoma, TCGA-KIRC: kidney renal clear cell carcinoma, TCGA-NBL: neuroblastoma, TCGA-TGCT: testicular germ cell tumors, TCGA-ACC: adrenocortical carcinoma, TCGA-THYM: thymoma, TCGA-PAAD: pancreatic adenocarcinoma, TCGA-SKCM: skin cutaneous melanoma, TCGA-BLCA: bladder urothelial carcinoma, TCGA-AML: acute myeloid leukemia, TCGA-MESO: mesothelioma, TCGA-BRCA: breast-invasive carcinoma, TCGA-LGG: brain lower-grade glioma, TCGA-UCEC: uterine corpus endometrial carcinoma, TCGA-SARC: sarcoma, TCGA-DLBC: lymphoid neoplasm diffuse large B-cell lymphoma, TCGA-UCS: uterine carcinosarcoma, TCGA-LUAD: lung adenocarcinoma, TCGA-OV: ovarian serous cystadenocarcinoma, TCGA-GBM: glioblastoma multiforme, TCGA-CESC: cervical squamous cell carcinoma and endocervical adenocarcinoma, TCGA-LUSC: lung squamous cell carcinoma, TCGA-READ: rectum adenocarcinoma, TCGA-WT: high-risk Wilms tumor, TCGA-STAD: stomach adenocarcinoma, TCGA-RT: rhabdoid tumor, TCGA-COAD: colon adenocarcinoma, TCGA-ESCA: esophageal carcinoma, and TCGA-HNSC: head and neck squamous cell carcinoma. **(D)** Prognosis of the metabolic score in the 36 TCGA projects. Hazard ratio with 95% CI and *p* values calculated using the inner cohort median values as the cutoff are visualized.

To confirm the reliability of the metabolic score model, another two independent HCC cohorts were used for validation (Figure 6A and Figure 6B). For the two HCC validation cohorts, namely, GSE14520 and GSE76427, only tumor tissues were used for validation. Using the median value as the cutoff, similar results, i.e., that high metabolic score HCC patients harbored unfavorable overall survival, were validated in both cohorts, revealing the metabolic score as a reliable tool for prognosis prediction (Figure 6A and Figure 6B). Other TCGA cohorts were also used to investigate the application of the metabolic score model (Figure 6C). The results showed good performance for other kinds of tumors of digestive or metabolic organs, such as kidney chromophobe (TCGA-KICH), kidney renal papillary cell carcinoma (TCGA-KIRP), kidney renal clear cell carcinoma (TCGA-KIRC), adrenocortical carcinoma (TCGA-ACC), pancreatic adenocarcinoma (TCGA-PAAD), and uterine corpus endometrial carcinoma (TCGA-UCEC) (*p* value < 0.05, Figure 6D).

## Discussion

There are multiple factors that are associated with the overall survival of HCC patients. Among the clinical characteristics of HCC patients, the tumor stage (AJCC stage) is the most relevant to the overall survival of HCC and the most commonly used. However, the AJCC stage only includes tumor characteristics but lacks information about the biological characteristics of HCC, such as molecular, metabolic, and immunologic features (Chidambaranathan-Reghupaty et al., 2021). Targeting metabolism has brought us new insights into cancer therapy. To provide enough energy for malignant proliferation and metastasis, some metabolic pathways are aberrantly altered in tumor tissues (DeBerardinis and Chandel, 2016). The tumor microenvironment is a mixture of tumor cells, stromal cells, and immune cells (Zheng et al., 2017). Abnormal cancer metabolism, such as glycolysis, plays important roles in drug resistance and the stemness of cancer cells (Park et al., 2020). Previous studies have reported high consistency between gene expression and protein levels and other kinds of omics (Gao et al., 2019), indicating that RNA sequencing data can be used to estimate the altered metabolic pathways in cancer research. Therefore, discovering abnormal metabolic pathways and targeting metabolism using RNA sequencing has brought new insights into cancer therapy (Luengo et al., 2017).

In this study, focusing on aberrantly expressed metabolic genes, we built a metabolic score model to predict the prognosis of HCC. Six metabolic-related genes were calculated as independent prognostic factors. Among the six metabolic genes, *LDHA* catalyzes the conversion of pyruvate and participates in the TCA cycle and has been reported to associate with tumor growth, maintenance, and invasion of HCC (Sheng et al., 2012; Miao et al., 2013). In the protein–protein interaction analysis, LDHA also acts as a hub gene that directly correlates with *HIF1A*, *EP300*, *TP53*, *PKM*, and other genes that are enrolled in metabolic pathways (Supplementary Figure S5). *FTCD* plays a role as a tumor suppressor gene in HCC and is critical for the catabolism of histidine (Chen et al., 2022). The expression level of histidine metabolism is also associated with the overall survival of HCC in our analysis. Several important pathways, including the TCA cycle and histidine metabolism, were key regulators in HCC progression. More evidence and experimental validation would be utilized to discover the mechanisms of these pathways in future work. In our project, using LASSO and Cox proportional hazards model, a six-gene-based metabolic model was constructed and relevant to the metabolic level and prognosis of HCC. Patients with higher scores had poorer prognoses. For patients with higher scores, pathways involved the cell cycle and tumorigenesis signaling pathways, such as TP53 signaling, indicating an exclusive correlation between TP53 and metabolism. Therefore, for patients with higher metabolic scores, TP53 signaling may be a valuable target for future analysis.

However, there are some limitations in our study. First, the potential mechanisms of metabolic pathways in overall survival need to be further explored. Next, further validation of the metabolic score model is needed, especially in clinical applications. Third, the key hepatocarcinogenesis mechanism for the metabolic score and potential therapeutic targets for patients with higher scores should be deeply investigated. In general, the six-gene-based metabolic score model, as an independent prognostic indicator of the overall survival of HCC patients, may help predict the procession of survival and provide insights for a metabolic analysis of cancer research.

## Conclusion

By comparing the expression profile of metabolic genes and pathways between tumor tissues and NAT, we found that HCC patients harbored lower expression levels of most metabolic pathways. The expression levels of several metabolic pathways were also correlated with the prognosis of HCC. To associate metabolic level with prognosis, a metabolic score model was built to predict the prognosis of overall survival of HCC based on the expression profile of dysregulated metabolic genes. Through validation using external independent cohorts, we believe that this six-gene-based metabolic score will be beneficial for prognosis prediction and the identification of potential therapeutic drug targets of HCC in the future.

## Data Availability

The datasets presented in this study can be found in online repositories. The names of the repository/repositories and accession number(s) can be found in the article/Supplementary Material.
