# Erratum: A metabolism- associated gene signature for prognosis prediction of hepatocellular carcinoma

**DOI:** 10.3389/fmolb.2023.1175415

**Published:** 2023-03-09

**Authors:** Frontiers Production Office

**Affiliations:** Frontiers Media SA, Lausanne, Switzerland

**Keywords:** hepatocellular carcinoma, metabolic pathways, metabolism-associated genes, LASSO model, prognosis signature

Due to a typesetting error, a symbol indicating **Equal contribution** was missing for authors Yilin Tian and Jing Lu. As a result, the following sentence was left out from the article:

“These authors have contributed equally to this work”.

The publisher apologizes for this mistake. The original version of this article has been updated.

