# Supplementary material for: A metabolism-associated gene signature for prognosis prediction of hepatocellular carcinoma
Source: Front Mol Biosci. 2022 Sep 30;9:988323. doi: 10.3389/fmolb.2022.988323 (PMC9561844; doi:10.3389/fmolb.2022.988323)
Supplement: Supplementary file 3 [file Table1.DOCX]

Supplementary Material

# Supplementary Figures


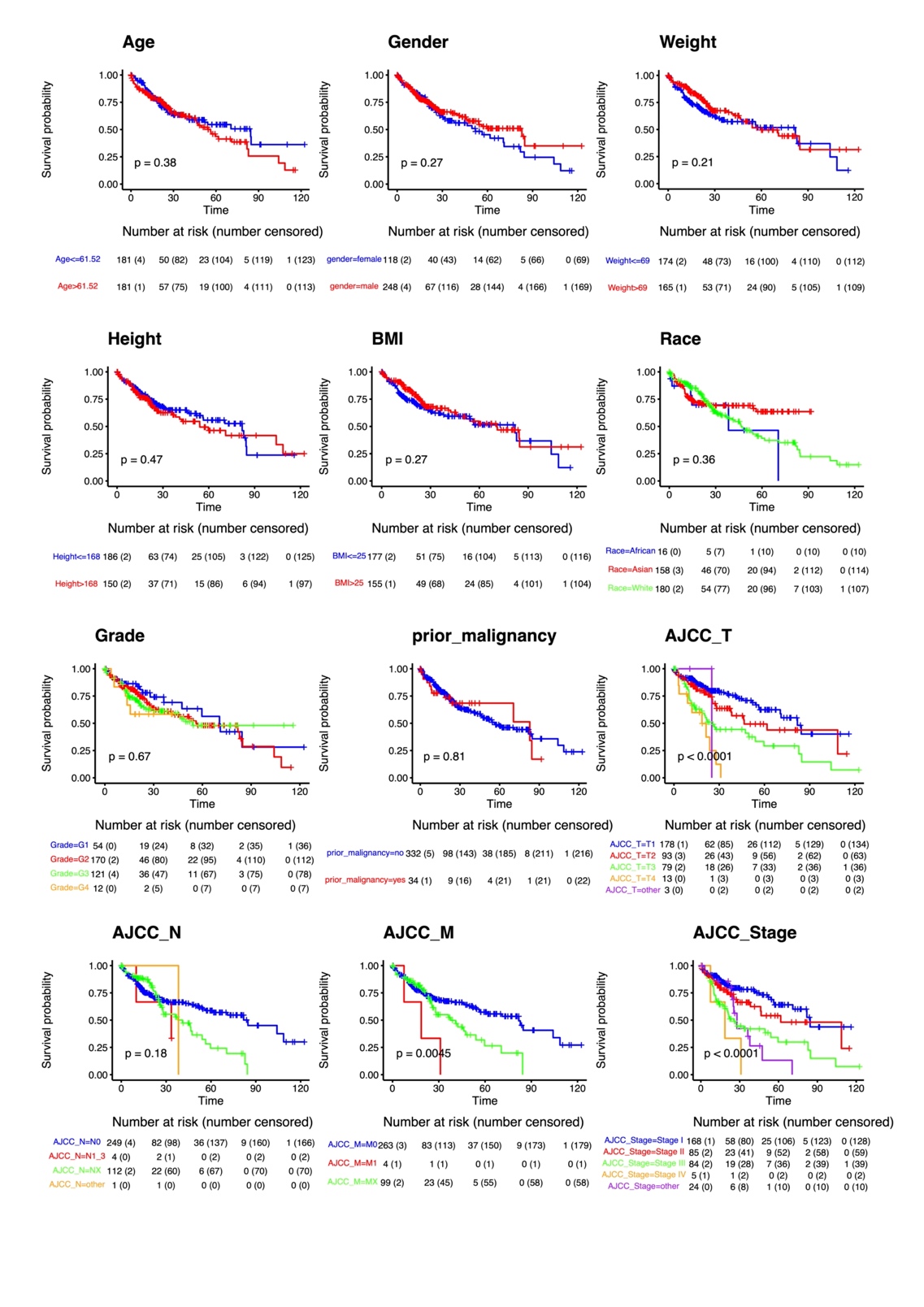


**Supplementary Figure 1.** Kaplan-Meier curves comparing overall survival of different clinical characteristics of HCC. Patients are separated into different groups according to the clinical characteristics. *P*-value is calculated using the log-rank test


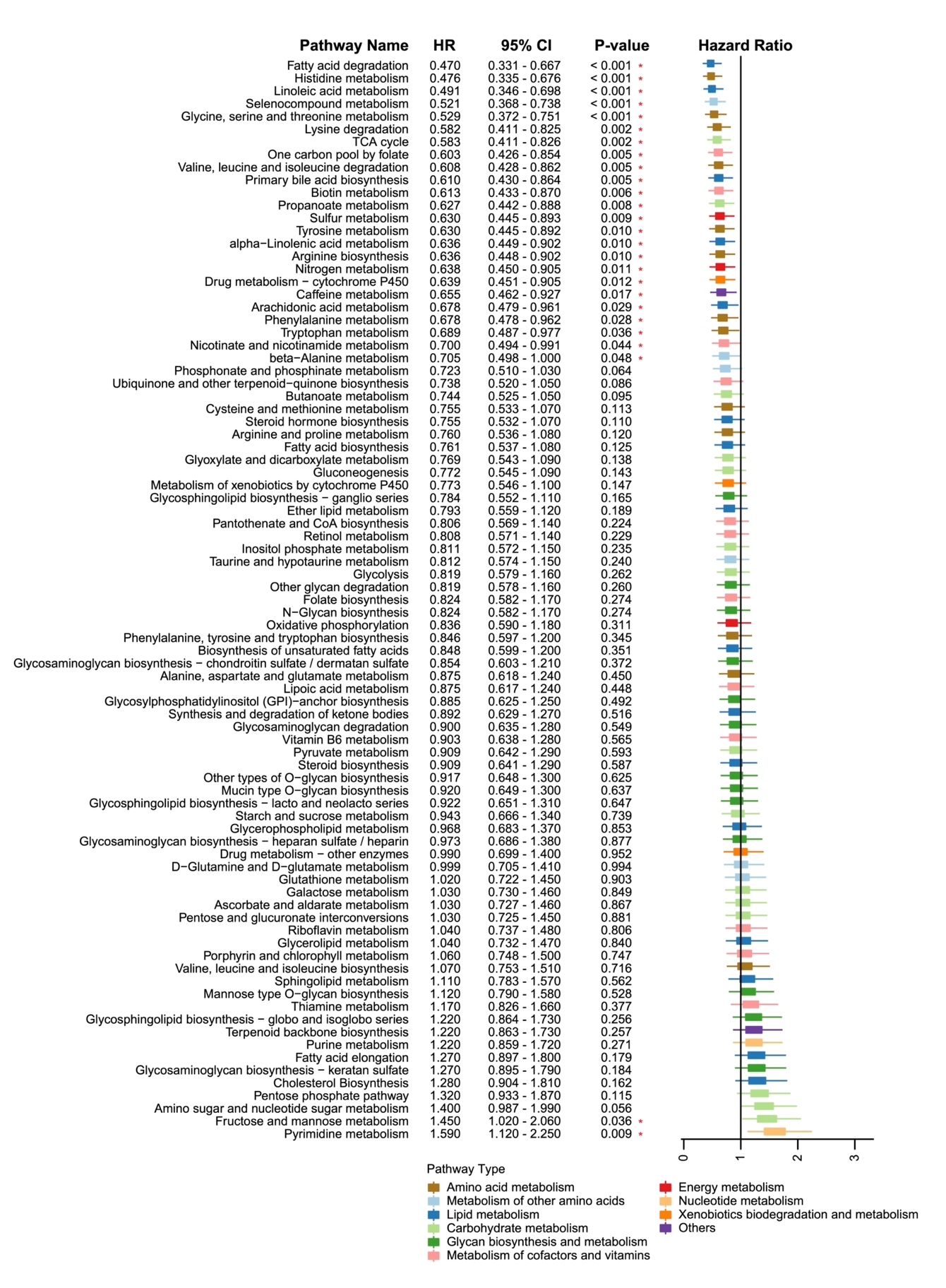


**Supplementary Figure 2.** Prognosis of the enrichment scores calculated using the 85 metabolic pathways in the HCC cohort. Cutoff used was the median value of each pathway.


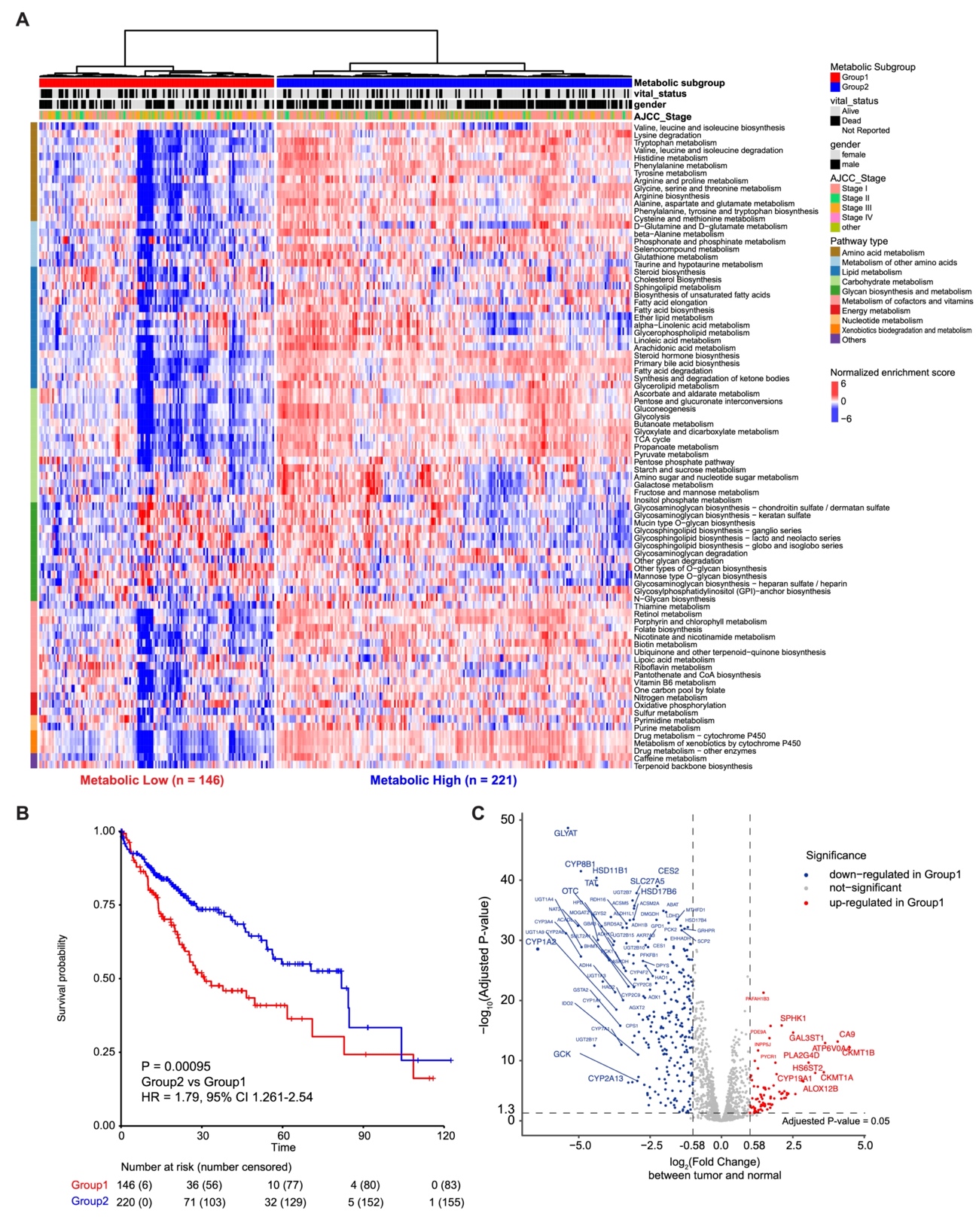


**Supplementary Figure 3.** (**A**) Heatmap of unsupervised hierarchical clustering using the 85 metabolic pathways. Each column represents one patient, and each row represents one pathways. Red color indicates higher expression level of the pathways and blue color indicates lower. (**B**) Kaplan-Meier curve of the two clusters generated from unsupervised hierarchical clustering of the 85 metabolic pathways. (**C**) Volcano plot of differentially expressed metabolic genes between HCC and NAT. Each dot represents one gene, and red dot represents this gene is significantly up-regulated in HCC, and blue dot represents down-regulated.


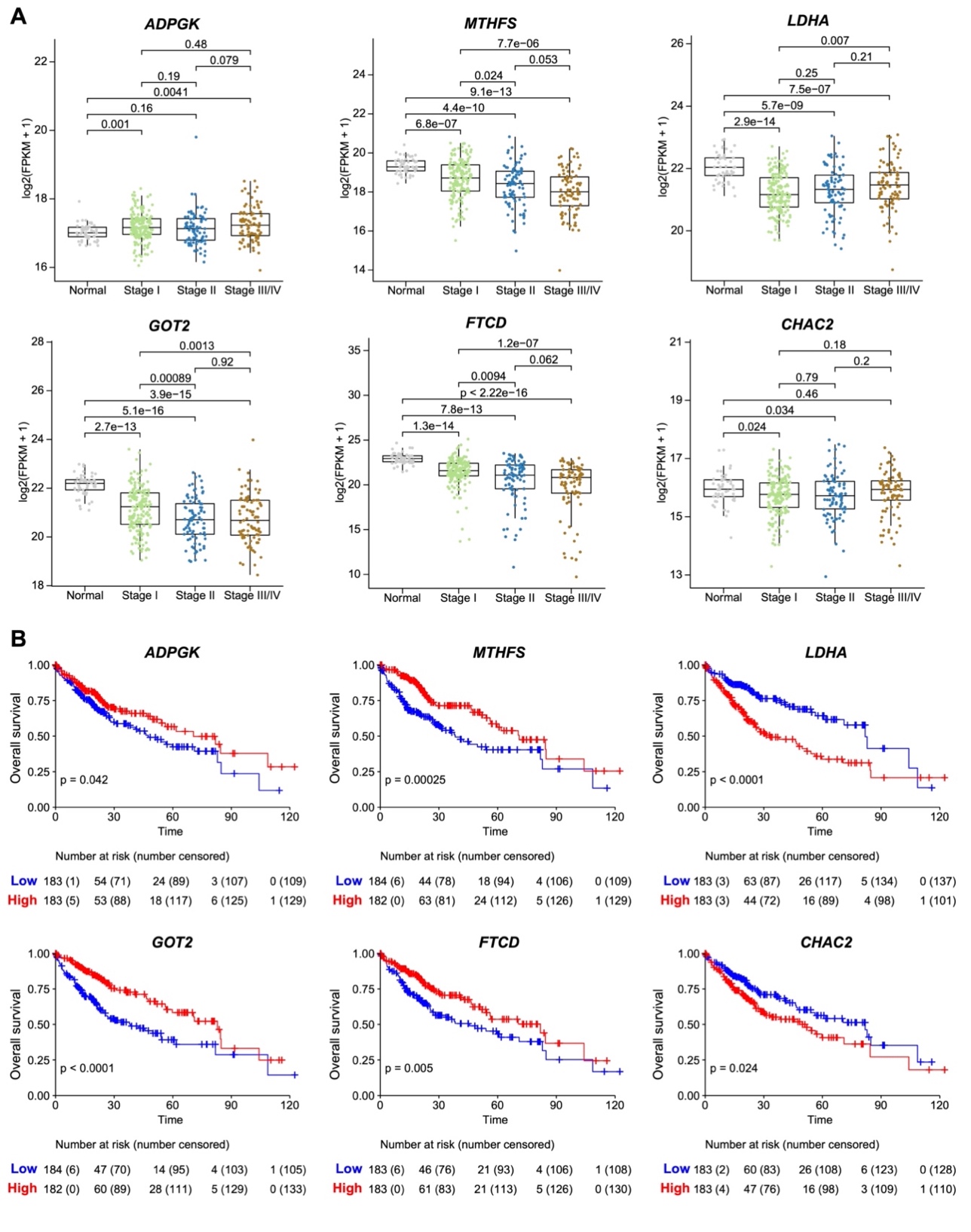


**Supplementary Figure 4. (A)** Boxplot of different gene expression in HCC with different tumor stages and NAT in TCGA cohort. *P*-values were calculated using Wilcoxon rank sum test (**B**) Kaplan-Meier curves of comparing overall survival of different gene expression level. Patients are separated into different groups according to the median value of gene expression. *P*-value is calculated using the log-rank test


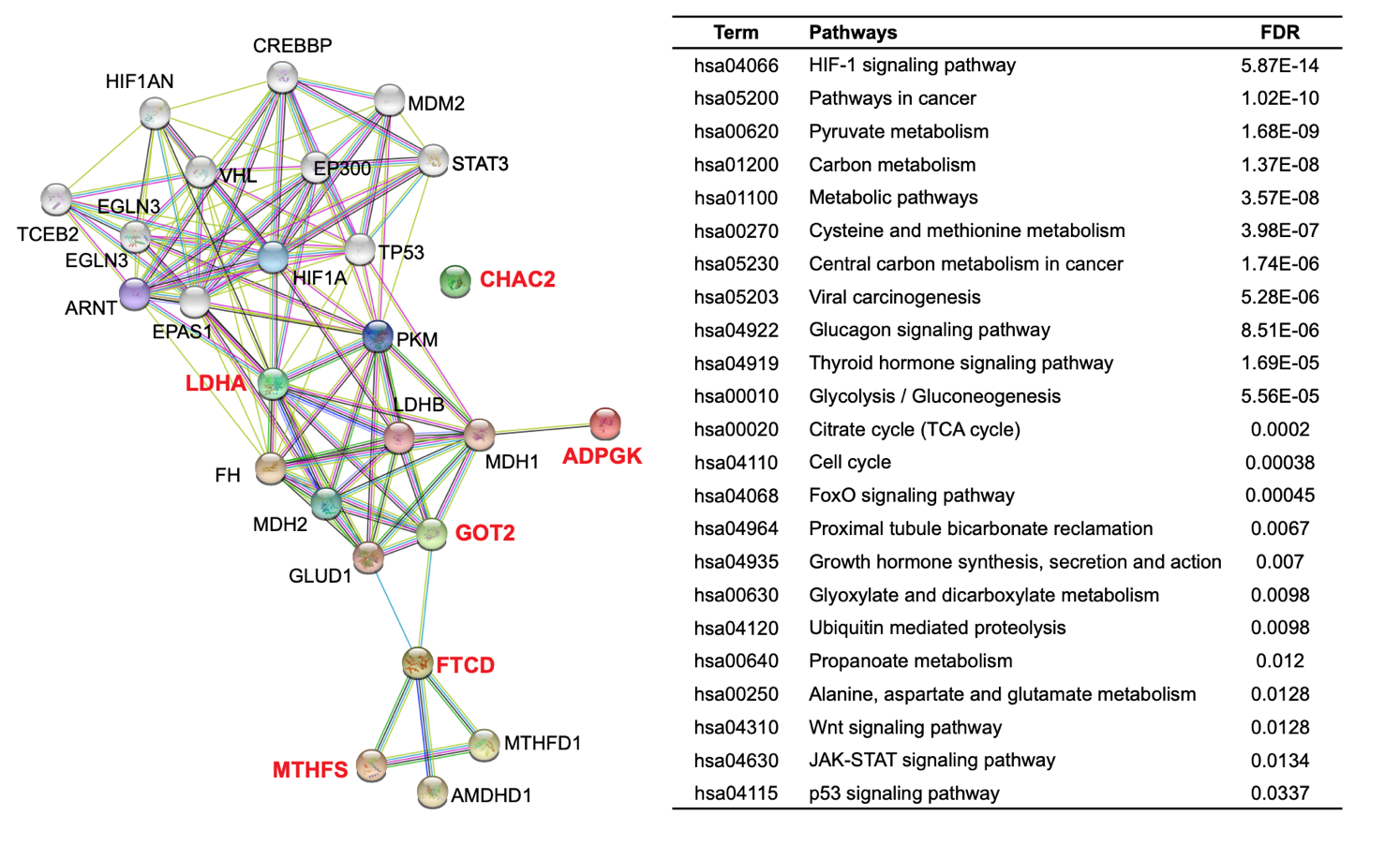


**Supplementary Figure 5.** Protein-protein interaction networks functional enrichment analysis using the six metabolic genes using STRING database.
